# Visual fields in patients who have undergone vitrectomy for complications of diabetic retinopathy. A prospective study

**DOI:** 10.1186/1471-2415-6-5

**Published:** 2006-01-26

**Authors:** Allon Barsam, Alistair Laidlaw

**Affiliations:** 1 Department of Ophthalmology, St Thomas' Hospital, Lambeth Palace Road, SE1 7EH, London; 2 Consultant Ophthalmologist, Department of Ophthalmology, St Thomas' Hospital, Lambeth Palace Road, SE1 7EH, London

## Abstract

**Backround:**

To determine the extent of visual field loss in patients who had required a pars plana vitrectomy secondary to complications of proliferative diabetic retinopathy.

**Methods:**

Patients that had undergone a vitrectomy on at least one eye for treatment of either vitreous haemorrhage or tractional retinal detachment were selected for study. ETDRS acuity and Humphrey binocular Esterman visual field testing were performed and compared to the minimum standards for safe driving as defined by the Royal College of Ophthalmologists in 1999. In addition to this Goldman kinetic visual fields using a III4e and V4e stimulus size and central 24-2 threshold test with the SITA-fast strategy were performed on the vitrectomised eye.

**Results:**

20 patients (n = 20) were recruited. Mean visual acuity in the eye being tested was 0.20 (Snellen 6/9.5). Results from the Humphrey field analyzer showed a mean number of abnormal stimulus locations of 71.2% (p < 0.005). 70% of patients had sufficient binocular acuity to drive and of these 71.4% were shown not to have a minimum visual field for safe driving on binocular Esterman field analysis.

**Conclusion:**

Vitrectomy potentially allows retention/restoration of good visual acuity in patients with complications of proliferative diabetic retinopathy. However patients may be suffering from unrecognized visual impairment consequent upon extensive visual field loss which in over two thirds of patients may be sufficiently severe to preclude safe driving.

## Backround

The necessity for a patient with proliferative diabetic retinopathy(PDR) to undergo a pars plana vitrectomy(PPV) either as a result of vitreous haemorrhage(VH)[1] or tractional retinal detachment (TRD) can be seen as a clinical end point of severe proliferative diabetic retinopathy. This group of patients will have inevitably suffered retinal damage due to a combination of retinal ischaemia[2,3] and panretinal laser photocoagulation(PRP)[4-13]. When these patients are assessed clinically, much emphasis is placed on their Snellen visual acuity as the main index of their visual function. This clearly ignores the functionally important aspect of visual fields. There have been many studies looking at the effect of PRP on reducing visual fields[4-13], however patients that have suffered VH or TRD are often excluded from such studies. It is thus possible that field loss is under appreciated in this group of patients.

Using three main perimetric testing techniques and standardized field analysis our study aimed firstly to increase our understanding of visual function and in particular visual fields in patients who have undergone a PPV for complications of PDR. Secondly, our study aimed to appreciate the impact of this visual field loss in terms of the ability of these patients to satisfy the UK minimal legal driving standard. Our study did not aim to distinguish between the various retinal insults that contribute to field loss in this group of patients, namely; PDR, PRP and the PPV itself [2-13].

## Methods

Consecutive patients who had undergone a pars plana vitrectomy (PPV) for treatment of haemorrhagic or tractional complications of proliferative diabetic retinopathy (PDR) over a six month period were selected by case note review. Any patient shown to have signs of suspicion for other causes of visual field loss, in particular glaucoma was excluded. Log minimal angle of resolution visual acuity(logMAR VA) was measured using an ETDRS test chart. The operated eye, or in the cases where both eyes had undergone surgery, the eye with the better acuity was selected for monocular visual field testing. Monocular Goldman kinetic visual fields using a standard Goldman bowl perimeter with stimulus sizes of III4e (10,000 Asb, 0 dB, 4 mm^2^) and V4e (10,000 Asb, <0 dB, 64 mm^2^) and monocular central 24-2 threshold test using a Humphrey visual field analyzer (HFA) with the SITA-fast strategy[14] were performed as well as binocular Esterman fields[15]. In each case appropriate near correction was employed in presbyopic patients. Fifteen age-gender matched healthy individuals additionally underwent Goldman field analysis for later quantitative comparison using the same test protocol.

The Goldman visual fields were cut out and weighed on electronic scales accurate to 0.0001 g in order to produce a quantitative representation of the overall field area. This was done for both III4e and V4e respectively.

Humphrey 24-2 monocular sensitivity loss was measured in terms of the number of stimulus locations with statistical probability levels of p less than 0.005. The count of abnormal stimulus locations (i.e. those with a SITA analysis statistical significance level of p < 0.005) was undertaken for the complete HFA program 24-2 field. Only clusters of three or more abnormal stimulus locations were included in the count, that is, a conservative criterion of abnormality was used thereby minimizing type 1 experimental error[10]. Mean deviation was also calculated for the Humphrey fields. Further visual field assessment was conducted using the binocular Esterman program of the Humphrey visual field analyzer. These Esterman fields were examined using the 1999 Royal College of Ophthalmologists definition for the minimum field for safe driving for group 1 ordinary drivers which states that 'the minimum visual field for safe driving is a field of vision of at least 120° on the horizontal meridian. In addition there should be no significant field defect in the binocular field which encroaches within 20° of fixation either above or below the horizontal meridian'[16]. Significant and therefore unacceptable central defects were defined by the current driver and vehicle licensing agency (DVLA) guidelines as 1) a cluster of four or more contiguous points that lies either wholly or partly within the central 20° area and 2) loss consisting of both a single cluster of 3 contiguous missed points and any additional missed points within the central 20° area.

The binocular BCVA was assessed for safe driving using the Royal College of Ophthalmologists' definition of 'approximately 6/10 on the Snellen chart'(logMAR equivalent 0.22). Only patients satisfying this requirement proceeded to have Esterman analysis. Any field test considered to be unreliable, for example due to a high number of fixation losses or false positive or negative errors, was repeated. This was only necessary for three patients and all three provided reliable fields on retesting.

## Results

20 patients were recruited (n = 20) with a mean age of 50.8 years (Range 29–73 years); 55% were male. In 55% of patients the diabetes was type 1; in the remaining 45% it was type 2. The mean duration of diabetes was 24.3 years (range 11–43). Indications for PPV were as follows; 30% for TRD, 55% for VH and the remaining 15% for both TRD and VH combined. All patients had in the past received at least one session of panretinal photocoagulation (PRP) to both eyes. Mean VA in the eye being tested was 0.20 logMAR (Snellen equivalent 6/9.5) with a range of VA from -0.1 to 0.7 logMAR. Of the 20 patients, 13 (65%) had a PPV and endolaser during vitrectomy on the eye being tested and these 13 patients underwent Goldman visual field and Humphrey field analysis.

The mean weight of the cut out III4e Goldman visual fields of the study patients was 0.5 g (range 0.1–1.1 g). The healthy individuals' cut out Goldman fields showed a mean weight of 1.3 g (range 1.3–1.4 g) at the III4e isopter. Thus our patients had a III4e isopter which was 38% that of healthy individuals (range 8 – 85%). At the V4e isopter, study patients had a mean cut out Goldman visual field weight of 0.8 g (range 0.2–1.4 g). Healthy individuals at the V4e isopter had a mean cut out visual field weight of 1.7 g (range 1.6–1.7 g). Thus our patients had a V4e isopter which was 49% that of healthy individuals (range 12 – 82%).

Results from the Humphrey field analyzer showed a mean number of abnormal stimulus locations of 37 out of a total of 52, i.e. 71.2% (range 44% to 94%). The average mean deviation of all field plots was -16.02 dB (p < 0.005) ; range -8.58 to -24.73 dB.

70% of patients had sufficient binocular acuity to drive and of these 71.4% were shown not to have a minimum visual field for safe driving on binocular Esterman field analysis. Of this 70% the mean binocular BCVA was 0.08 LogMAR (Snellen 6/7.5+1) with a range of -0.1 to 0.22 LogMAR. The mean BCVA of the weaker eye was 0.48 LogMAR (snellen 6/19+1) with a range of -0.18 to 1.0 LogMAR.

## Discussion

It is important for Ophthalmologists to recognize the extent of visual impairment suffered by patients with severe proliferative diabetic retinopathy. Our study shows the global nature of visual field loss in such patients. The study patients had on average lost 62% and 51% of their Goldman III4e and V4e visual field areas respectively and using our conservative definition of three missed points 71% of their central 24 degrees vision on HFA analysis. Only 20% were deemed visually fit to drive using current DVLA acuity and visual field guidelines. This is despite an average log MAR visual acuity of 0.20 (Snellen equivalent 6/9.5).

Our method of quantifying Goldman visual fields by cut out weight has not to our knowledge been reported in the literature. We used uniform and identical paper on which to plot the Goldman fields and thus the cut out weight of the visual field gives an accurate representation of the overall area of the visual field.

Our rate of failure of Esterman fields for safe driving is similar to original reports showing that 80% of patients who have had bilateral PRP with a 500 micron burn for PDR will fail the current DVLA field test[17]. Subsequent reports utilizing smaller burns showed that fewer patients fail the test[18].

Vitrectomy potentially allows retention/restoration of good acuity in patients with complications of PDR. However patients may be suffering from unrecognized visual impairment consequent upon extensive visual field loss which in 75% of patients is sufficiently severe to preclude safe driving.

Limitations of our study include a small sample size as well as a difficulty in determining accurately from the notes the location and extent of PRP that these patients had in the past. Our study group may also have been biased towards those with a good acuity outcome. Static perimetry was not performed as part of the monocular Goldman fields and thus our weighting method may overestimate field survival due to the probability of scotomas within the peripheral isopters.

We recommend that diabetic patients who require vitrectomy secondary to complications of proliferative retinopathy are advised that their peripheral vision is likely to have been greatly reduced and that they are unlikely to be able to fulfill current UK driving regulations.

## Competing interests

The author(s) declare that they have no competing interests.

## Authors' contributions

AB: First author. Applied for ethics approval, recruited patients, conducted visual field testing. Drafted manuscript

DAHL: Second author. Conceived of the study. Designed study. Helped draft the manuscript

Both authors have read and approved the final manuscript

## Pre-publication history

The pre-publication history for this paper can be accessed here:


